# Supplementary material for: Effectiveness of suvorexant versus benzodiazepine receptor agonist sleep drugs in reducing the risk of hip fracture: Findings from a regional population-based cohort study
Source: PLoS One. 2023 Apr 24;18(4):e0284726. doi: 10.1371/journal.pone.0284726 (PMC10124872; doi:10.1371/journal.pone.0284726)
Supplement: S1 Table — (DOCX) [file pone.0284726.s001.docx]

**S1 Table. Sleep drugs included in each drug category**

| **Sleep drug categories** | **Sleep drugs** |
| --- | --- |
| Benzodiazepines | Estazolam, flurazepam, nitrazepam, haloxazolam, triazolam, flunitrazepam, brotizolam, lormetazepam, oxazolam, cloxazolam, clorazepate, diazepam, fludiazepam, bromazepam, medazepam, lorazepam, alprazolam, flutazolam, mexazolam, flutrazepam, chlordiazepoxide, loflazepate, quazepam, rilmazafone |
| Benzodiazepine receptor agonists | Zolpidem, zopiclone, eszopiclone |
| Orexin receptor agonist | Suvorexant |
