# Supplementary material for: Effectiveness of suvorexant versus benzodiazepine receptor agonist sleep drugs in reducing the risk of hip fracture: Findings from a regional population-based cohort study
Source: PLoS One. 2023 Apr 24;18(4):e0284726. doi: 10.1371/journal.pone.0284726 (PMC10124872; doi:10.1371/journal.pone.0284726)
Supplement: S2 Table — ICD-10: International Classification of Diseases, 10th Revision, AIDS: acquired immunodeficiency syndrome, HIV: human immunodeficiency virus. (DOCX) [file pone.0284726.s002.docx]

**S2 Table. Definitions of comorbidities using** **ICD-10 codes**

| **Comorbidity** | **ICD-10 code** |
| --- | --- |
| Cardiac arrhythmias | I441, I442, I443, I456, I459, I47, I48, I49, R000, R001, R008, T821, Z450, Z950 |
| Valvular disease | A520, I05, I06, I07, I08, I091, I098, I34, I35, I36, I37, I38, I39, Q230, Q231, Q232, Q233, Z952, Z953, Z954 |
| Pulmonary circulation disorders | I26, I27, I280, I288, I289 |
| Hypertension uncomplicated | I10 |
| Hypertension complicated | I11, I12, I13, I15 |
| Other neurological disorders | G10, G11, G12, G13, G20, G21, G22, G254, G255, G312, G318, G319, G32, G35, G36, G37, G40, G41, G931, G934, R470, R56 |
| Diabetes uncomplicated | E100, E101, E109, E110, E111, E119, E120, E121, E129, E130, E131, E139, E140, E141, E149 |
| Diabetes complicated | E102, E103, E104, E105, E106, E107, E108, E112, E113, E114, E115, E116, E117, E118, E122, E123, E124, E125, E126, E127, E128, E132, E133, E134, E135, E136, E137, E138, E142, E143, E144, E145, E146, E147, E148 |
| Hypothyroidism | E00, E01, E02, E03, E890 |
| Renal failure | I120, I131, N18, N19, N250, Z490, Z491, Z492, Z940, Z992 |
| Liver disease | B18, I85, I864, I982, K70, K711, K713, K714, K715, K717, K72, K73, K74, K760, K762, K763, K764, K765, K766, K767, K768, K769, Z944 |
| Peptic ulcer disease, excluding bleeding | K257, K259, K267, K269, K277, K279, K287, K289 |
| Lymphoma | C81, C82, C83, C84, C85, C88, C96, C900, C902 |
| Solid tumor without metastasis | C00, C01, C02, C03, C04, C05, C06, C07, C08, C09, C10, C11, C12, C13, C14, C15, C16, C17, C18, C19, C20, C21, C22, C23, C24, C25, C26, C30, C31, C32, C33, C34, C37, C38, C39, C40, C41, C43, C45, C46, C47, C48, C49, C50, C51, C52, C53, C54, C55, C56, C57, C58, C60, C61, C62, C63, C64, C65, C66, C67, C68, C69, C70, C71, C72, C73, C74, C75, C76, C97 |
| Rheumatoid arthritis/collagen | L940, L941, L943, M05, M06, M08, M120, M123, M30, M310, M311, M312, M313, M32, M33, M34, M35, M45, M461, M468, M469 |
| coagulopathy | D65, D66, D67, D68, D691, D693, D694, D695, D696 |
| Metastatic cancer | C77, C78, C79, C80 |
| Obesity | E66 |
| Weight loss | E40, E41, E42, E43, E44, E45, E46, R634, R64 |
| Fluid and electrolyte disorders | E222, E86, E87 |
| Deficiency anemia | D508, D509, D51, D52, D53 |
| Alcohol abuse | F10, E52, G621, I426, K292, K700, K703, K709, T51, Z502, Z714, Z721 |
| Drug abuse | F11, F12, F13, F14, F15, F16, F18, F19, Z715, Z722 |
| Depression | F204, F313, F314, F315, F32, F33, F341, F412, F432 |
| Psychoses | F20, F22, F23, F24, F25, F28, F29, F302, F312, F315 |
| AIDS/HIV | B20, B21, B22, B24 |
| Congestive heart failure | I99, I110, I130, I132, I255, I420, I425, I426, I427, I428, I429, I43, I50, P290 |
| Peripheral vascular disorders | I70, I71, I731, I738, I739, I771, I790, I792, K551, K558, K559, Z958, Z959 |
| Chronic pulmonary disorders | I278, I279, J40, J41, J42, J43, J44, J45, J46, J47, J60, J61, J62, J63, J64, J65, J66, J67, J684, J701, J703 |
| Paralysis | G041, G114, G801, G802, G81, G82, G830, G831, G832, G833, G834, G839 |
| Blood loss anemia | D500 |

AIDS: acquired immunodeficiency syndrome, HIV: human immunodeficiency virus, ICD-10: International Classification of Diseases, 10th Revision.
